# Am I Self-Conscious? (Or Does Self-Organization Entail Self-Consciousness?)

**DOI:** 10.3389/fpsyg.2018.00579

**Published:** 2018-04-24

**Authors:** Karl Friston

**Affiliations:** Wellcome Trust Centre for Neuroimaging, Institute of Neurology, University College London (UCL), London, United Kingdom

**Keywords:** active inference, predictive processing, variational, free energy, Bayesian, model selection, dynamics

## Abstract

Is self-consciousness necessary for consciousness? The answer is yes. So there you have it—the answer is yes. This was my response to a question I was asked to address in a recent AEON piece (https://aeon.co/essays/consciousness-is-not-a-thing-but-a-process-of-inference). What follows is based upon the notes for that essay, with a special focus on self-organization, self-evidencing and self-modeling. I will try to substantiate my (polemic) answer from the perspective of a physicist. In brief, the argument goes as follows: if we want to talk about creatures, like ourselves, then we have to identify the characteristic behaviors they must exhibit. This is fairly easy to do by noting that living systems return to a set of attracting states time and time again. Mathematically, this implies the existence of a Lyapunov function that turns out to be model evidence (i.e., self-evidence) in Bayesian statistics or surprise (i.e., self-information) in information theory. This means that all biological processes can be construed as performing some form of inference, from evolution through to conscious processing. If this is the case, at what point do we invoke consciousness? The proposal on offer here is that the mind comes into being when self-evidencing has a temporal thickness or counterfactual depth, which grounds inferences about the consequences of *my* action. On this view, consciousness is nothing more than inference about *my* future; namely, the self-evidencing consequences of what I could do.

## Introduction

There are many phenomena in the natural sciences that are predicated on the notion of “self”; namely, self-information, self-organization, self-assembly, self-evidencing, self-modeling, self-consciousness and self-awareness. To what extent does one entail the others? This essay tries to unpack the relationship among these phenomena from first (variational) principles. Its conclusion can be summarized as follows: living implies the existence of “lived” states that are frequented in a characteristic way. This mandates the optimization of a mathematical function called “surprise” (or self-information) in information theory and “evidence” in statistics. This means that biological processes can be construed as an inference process; from evolution through to conscious processing. So where does consciousness emerge? The proposal offered here is that conscious processing has a temporal thickness or depth, which underwrites inferences about the consequences of action. This necessarily lends inference a purposeful and self-evidencing aspect that has the hallmarks of consciousness. Finally, we will touch on the distinction between consciousness and self-consciousness; by asking whether self-consciousness only emerges when inferring or disambiguating the author of my sensations; in other words, “did I cause that or did you?”

Our starting point is to consider consciousness as a process—like the weather, evolution or optimization, as opposed to a state—like sleep—or a state of being. I find this perspective useful when thinking about consciousness. My favorite trick is to replace the word “consciousness” with “evolution” in any sentence to see if the sentence makes sense^1^. For example, the question:

“What is consciousness for?”

becomes:

“What is evolution for?”

If evolution is not “for” anything^2^ (Dennett, 2017), one can dismiss these questions as ill-posed or based on a category error (i.e., assigning an attribute to something that cannot possess that attribute). This substitution trick can sometimes be useful in organizing responses to well posed questions. For example:

“Are there different sorts of consciousness?”

Well yes, in that there are many evolutionary or natural selection processes that operate at different timescales—in the sense of Universal Darwinism (Campbell, 2016)—and contextualize each other: e.g., hierarchical co-evolution (Rosenman and Saunders, 2003), evolutionary psychology and beyond (Heyes and Frith, 2014). Notice I have slipped in “selection” as another process. In an evolutionary setting, selection brings with it notions like selection for selectability, otherwise known as second order selection (Kauffman, 1993; Woods et al., 2011). Immediately, this speaks to conscious processes that operate on conscious processes—a theme that we will develop later. Let us first establish a few ground rules about the nature of processes—and see how far one can get by applying those rules to consciousness.

## Processes, self-evidencing, and inference

On a physicists view, any (weakly mixing random dynamical) process can be completely characterized by a function of its current state. Formally speaking, the current state corresponds to a location in some abstract state space and the function is known as a *Lyapunov function* (i.e., a function of the states that always increases, on average: see Table 1). For people not familiar with Lyapunov functions, imagine the flow of water down a mountainside. The Lyapunov function enables one to predict the flow at any point on the landscape. In this case, the Lyapunov function corresponds to a gravitational potential that depends upon the height of the mountainside. These sorts of flow are often referred to as *gradient flows* (i.e., on the gradients established by the Lyapunov function). This gives rise to the appearance of some force (e.g., gravity) that provides a complementary description of—or explanation for—the flow. Exactly the same ideas apply in a more abstract and general setting, when considering the flow of any states that characterize a system. So what is the most general form of gravity for systems like you and me?

**Table 1:** Glossary of terms.

**Many of the terms used in Bayesian inference have formal meanings that sometimes depart from their folk psychology usage. Some common terms are listed below**.
**Active inference**: the minimization of variational free energy through approximate Bayesian inference and active sampling of (sensory) data. This active sampling itself induces posterior beliefs over action, under prior beliefs that action will minimize free energy in the future. This is equivalent to resolving uncertainty with epistemic, information-seeking behavior: see (Friston et al., 2015b) for details.
**(Bayesian) belief**: a probability distribution over a random variable, such as a latent cause or hidden state of the world causing (sensory) data.
**Consciousness**: the process of (approximate Bayesian) inference: see (Hobson and Friston, 2014) for details.
**Ergodicity**: the possession of measurable characteristics: a process is ergodic if its statistical properties can be deduced from a single, sufficiently long, random sample of the process. Typically, ergodic processes revisit states after a sufficient period of time.
**Generative model**: a probabilistic model, comprising a likelihood and prior beliefs that specifies how (sensory) consequences are generated by latent causes, such as hidden states and model parameters.
**Inference**: the optimization of beliefs by maximizing Bayesian model evidence or minimizing surprise. Approximate Bayesian inference corresponds to minimizing variational free energy.
**Interoceptive**: pertaining to internal (autonomic) states: see (Craig, 2013; Seth, 2013) for details.
**Likelihood**: the probability of observing (sensory) data given the causes of those data.
**Lyapunov function**: for a given non-linear dynamical system, a Lyapunov function is a positive definite scalar function that decreases along the trajectories of the system. Practically, it is generally used to establish a stability the system.
**(Bayesian) Model evidence**: the probability of (sensory) data under a generative model. Also known as the marginal likelihood. The log model evidence is approximated by (negative) variational free energy: see (Beal, 2003) for details.
**Prediction**: the prediction of (sensory) data based upon posterior beliefs about the causes of sensory consequences.
**Posterior belief**: a Bayesian belief after sampling (sensory) data.
**Prior constraint or belief**: a Bayesian belief prior to sampling (sensory) data.
**(Variational) Free energy**: a functional of sensory data and posterior beliefs. Free energy scores the surprise of (sensory) data, given posterior beliefs about how they were caused. This furnishes an approximation to model evidence.
**Surprise, surprisal or self-information**: the negative log probability of an event, under a generative model of the process producing that event.

We are only interested in one sort of system. These are processes where (the neighborhood of) certain states are re-visited time and time again; for example, the biological rhythms that characterize cardiorespiratory cycles—or the daily routine we enjoy every Monday, on getting up and going to work. These special (weakly mixing, weakly ergodic) processes—like ourselves—possess a Lyapunov function that is the (log) probability of being in any particular state. This means, on average, I must move toward states I am more likely to occupy. This may sound trivially simple but has enormous implications for the nature of any (interesting) process that possesses an attracting set of states.

In information theory, this Lyapunov function is called (negative) *self-information, surprisal* or, more simply, *surprise* (Jones, 1979). In statistics and machine learning, it is known as the *marginal likelihood* or Bayesian *model evidence*; namely, the probability of observing some states or data, given the process or model generating those states (Fox and Roberts, 2011). The important thing here is that surprise (respectively, evidence) characterizes the process because all the system's states change to minimize (respectively maximize) this quantity. So what does this mean? It means that any system that revisits a particular set of states will necessarily be engaged in the process of minimizing surprise or maximizing evidence. In short, all systems that exist (in the above sense) are *self-evidencing* (Hohwy, 2016). From the point of view of stochastic thermodynamics, the time average of this surprise is called *entropy* (Sekimoto, 1998; Seifert, 2012; Still et al., 2012). This means that self-evidencing processes, by definition, resist the second law of thermodynamics (that entails an increase in disorder or entropy)^3^. In short, they are living. This dynamical formulation means that we can interpret any system in complementary but equivalent ways; we can consider its statistical mechanics (e.g., information theory and thermodynamics) or appeal to a Bayesian mechanics (e.g., predictive processing). The two go hand-in-hand, thereby equipping any random dynamical system with a mechanics or physics that can be formulated in terms of Bayesian beliefs, inference and sentience.

The notions of a weakly mixing ergodic processes are relatively intuitive. For example, imagine I placed a drop of oil in a cup of water. I then came back and measured its size every 3 min, until I was satisfied I had an accurate estimate of its diameter. The very fact that the oil drop can be measured testifies to its ergodicity (i.e., the possession of measurable characteristics). Contrast this with a drop of ink in water. After 3 min, I come back and find I have nothing to measure. This is because the ink molecules have been dispersed throughout the water by random molecular fluctuations. In short, systems that do not possess an attracting set dissipate, disperse, decay or die. The notion of fluctuations and dissipation is at the heart of many fundamental theorems in statistical physics; including generalizations of the celebrated second law of thermodynamics (Evans and Searles, 1994; Evans, 2003; Seifert, 2012).

Clearly, a drop of oil is not a very interesting process. A more tangible and interesting example would be you; repeatedly visiting a small number of states as you rise in the morning, brush your teeth, have morning coffee, go to work, *etc*. You are you because you revisit (the neighborhood of) these attracting states time after time. Your life traces out a path on this delicately structured attracting set or manifold, where your highly convoluted orbits—or strange loops—keep bringing you back to where you once came from Hofstadter (2007). Technically speaking, you are a random dynamical attractor, with an attracting set of states that fills a large part of (state) space, yet has an intrinsically small volume (i.e., measure). In other words, of all possible states you could occupy, there are only a small number that you would characteristically be found in:

*“But, however many ways there may be of being alive, it is certain that there are vastly more ways of being dead, or rather not alive.”—*Richard Dawkins (Dawkins, 1996, p. 9)

With the notion of a random dynamical attractor, questions about the nature of life resolve into questions about the dynamics such systems must possess if they exist. In short, life is its own existence proof.

## Everything as inference

To the extent one accepts the above formulation; one has the ultimate deflationary account of everything (that exists). In other words, every process that can be measured (i.e., has characteristic states that are occupied repeatedly) must, in virtue of its existence, increase Bayesian model evidence^4^ (Schrödinger, 1944; Friston, 2013). In other words, all systems must necessarily behave in a way that increases the evidence for their own existence (Ramstead et al., 2017). In this sense, every biological process is quintessentially inferential. But does this make sense? One can hardly consider the process of evolution or natural selection in terms of inference—or can one? In fact, this is exactly the interpretation currently found in theoretical neurobiology (Campbell, 2016). In this setting, the sorts of equations used to describe natural selection turn out to be exactly the same sorts of processes used for data assimilation and Bayesian filtering. For example, the replicator equation is formally equivalent to a Kalman filter (Harper, 2011; Frank, 2012). Effectively, this means that natural selection is nature's way of performing Bayesian model selection; testing various hypotheses (i.e., models or phenotypes) and scoring them to select phenotypes that have the greatest evidence: adaptive fitness is just the evidence for the hypothesis that this phenotype can survive in this econiche.

**Figure 1:**
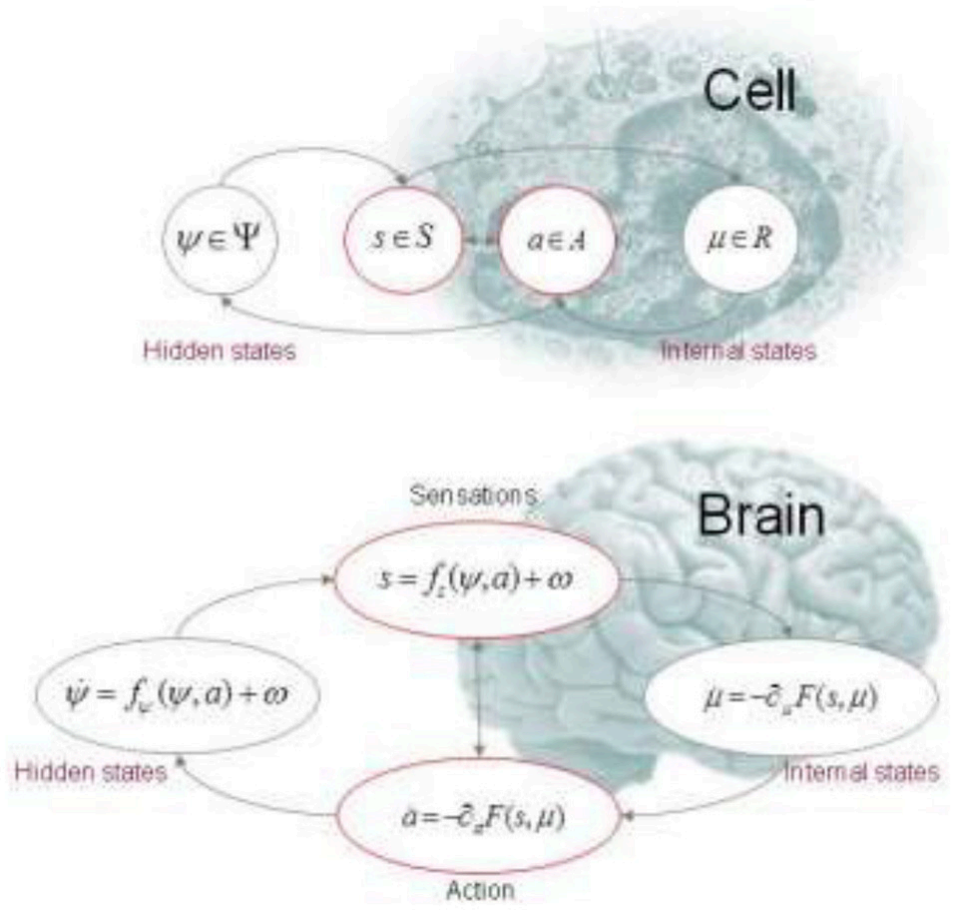
This figure illustrates the partition of states into internal and hidden or external states that are separated by a Markov blanket—comprising sensory and active states. The lower panel shows this partition as it would be applied to action and perception in the brain; where active and internal states minimize a free energy functional of sensory states. The ensuing self-organization of internal states then corresponds to perception, while action couples brain states back to external states. The upper panel shows exactly the same dependencies but rearranged so that the internal states are associated with the intracellular states of a cell, while the sensory states become the surface states of the cell membrane overlying active states (e.g., the actin filaments of the cytoskeleton).

This may appear to be an unnatural interpretation of natural selection; however, it is a mathematical truism that tells us that self-evidencing is just another way of describing adaptive biological systems. Applying the same argument to consciousness suggests that *consciousness must also be a process of inference*. This sounds more natural and starts to equip conscious processing with a mindful aspect. Taken literally—which many people do, in terms of the Bayesian brain hypothesis and predictive processing (Kersten et al., 2004; Clark, 2013)—it means that conscious processing is about inferring the causes of sensory states and navigating the world to elude surprises.

There is a vast amount of anatomical and physiological evidence in support of this notion. In other words, when one packs the imperative to minimize surprise, the resulting process theories offer a very plausible explanation for how our brains actually work. These include things like predictive coding, or more generally predictive processing (Bastos et al., 2012; Shipp et al., 2013; Shipp, 2016; Friston et al., 2017a). Furthermore, there are interesting arguments that rest upon hierarchical inference—that call on deep generative models—which speak to self-modeling and a possible metaphor for self-consciousness (Metzinger, 2003). In other words, parts of our brain (and body) could be construed as inferring a sensorium that is constituted by other parts of the brain (and body). However, we will leave these arguments aside for the moment and ask whether Bayesian mechanics is really a sufficient account of consciousness.

## Viruses and vegans

On the above account, any system or process that revisits characteristic states—known technically as the attracting set of a *random dynamical system* (Crauel and Flandoli, 1994; Arnold, 2003)—must, at some level, be inferring (i.e., modeling) the causes of its sensory impressions of the outside world (Friston, 2013). Does this mean that every sentient creature that possesses characteristic (attracting) states can be described as conscious? In other words, if one accepts that conscious processing is a process of inference; does all inference qualify as consciousness?

The deflationary account would suggest yes. For example, people already think of natural selection as a process of inference; namely, inference about the sorts of phenotypes a particular econiche is most apt to support (Frank, 2012). If we put a bit of circular causality into the mix (where the phenotype builds its econiche), one has a very plausible metaphor for embodied cognition, designer environments and many other aspects of the enactivist paradigm (Bruineberg and Rietveld, 2014). But is evolution really conscious?

Probably not, for the following reason: previously, we have noted selection rests on processes embedded at multiple hierarchical levels—Darwinism within Darwinism all the way down to the selection of dendritic spines on single neurons in our brain (Edelman, 1993; Kiebel and Friston, 2011). At what point do these deeply entailed and hierarchical selection processes qualify as conscious? For example, a virus possesses all the biotic, self-organizing and implicitly inferential dynamics to qualify as a process of Bayesian inference; however, it does not have the same qualities as a vegan. So what puts these two sorts of sentient creatures apart? The answer offered below brings us to self-consciousness.

## Thick and deep models^5^

The answer entertained here rests upon the two-way coupling between a system and the world. The world acts on the system—providing sensory impressions that form the basis of an implicit Bayesian inference, while the system acts upon the world to change the flow of sensations. Both must—in virtue of the system's existence—serve to minimize surprise (or maximize the evidence for the system). If action depends upon inference then systems can make inferences about (the consequences of) their action: however, there is an important twist here.

A living system cannot infer the consequences of its action unless it embodies a model of the future. This follows from the simple fact that the arrow of time requires the consequences of action to postdate action *per se*. This is important because it means that the (generative) models capable of inferring the consequences of action must necessarily endow inference with a *temporal thickness* (Chouraqui, 2011). In other words, the model or system must have an internal dynamics that has a mnemonic aspect; namely, the capacity to infer the past and future (i.e., to postdict and predict)^6^. Such generative models, necessary for planning, immediately confer an ability to not only represent the future but to represent the past. This follows because the current time must be located within the temporal span of the generative model, endowing the model with both a predictive (anticipatory) and postdictive capacity. This clearly has close relationships with the notion of mental time travel (Buckner and Carroll, 2007; Schacter et al., 2007) and a form of autonoetic memory for recently experienced events (Tulving, 2002) that are contextualized by subsequent evidence accumulation.

The following question now poses itself: if a system has a temporally thick generative model, what actions will it infer or select? The answer to this is simple and appeals to the deflationary account of self-evidencing above. Put simply, thick or deep generative models will minimize the surprise (i.e., maximize model evidence) expected following an action. The proof follows by *reductio ad absurdum*: Systems that select actions which do not minimize surprise cannot exist—because existence entails a minimization of surprise. So what does this mean heuristically?

The minimization of expected surprise through action (known as *active inference*) simply means we act to resolve uncertainty (Friston et al., 2015b). This follows from the fact (above) that expected surprise is entropy or uncertainty^7^. In short, deep models try to resolve uncertainty and avoid surprises in the future (like being cold, hungry, or dead). Note that surprise does not have any anthropomorphic or folk psychology meaning in this setting—it is just a way of labeling states that are characteristic of—or attract—the system in question. The second important aspect of these sorts of systems is that their action upon the world is endowed with a purpose. Furthermore, this purposeful and possibly mindful active inference has all the hallmarks of agency (i.e., the apparent capacity to act independently and to make choices in a way that is affected by belief structures formed through experience).

One could then describe systems that have evolved thick generative models (with deep temporal structure) as *agents*. It now seems more plausible to label these sorts of systems (agents) as conscious, because they have beliefs about what it is like to act; i.e., just be an agent. Furthermore, because active inference is necessarily system-centric the self-evidencing of motile creatures can only be elevated to self-consciousness if, and only if, they model the consequences of their actions. Put simply, this suggests that viruses are not conscious; even if they respond adaptively from the point of view of a selective process. Vegans, on the other hand, with deep (temporally thick) generative models are self-evidencing in a prospective and purposeful way, where agency and self become an inherent part of action selection. In a similar vein, we elude the problems of calling evolution conscious, because the process of natural selection minimizes surprise (i.e., maximizes adaptive fitness) but not expected surprise or uncertainty (i.e., adaptive fitness expected under alternative evolutionary operations or selection). The key difference between (self) consciousness and more universal processes then appears to be the locus of selection. In non-conscious processes this selection is realized in the here and now with selection among competing systems (e.g., phenotypes). Conversely, the sort of selection we have associated with (self) consciousness operates within the same system—a system that can simulate multiple futures, under different actions, and select the action that has the least surprising outcome.

Heuristically, the difference between thick and thin models is manifest in terms of the structure of the random dynamical attractor. A key aspect of this attracting manifold is the time elapsed between revisiting the same state (or neighborhood). This can be illustrated by contrasting me with a virus. I revisit the same states over very long time periods compared to a virus; for example, every morning I take my latte in the park outside my office—and every Christmas I attend midnight mass. The virus, on the other hand, is unlikely to be found celebrating its birthday on an annual basis—and indeed is unlikely to last that long. Again, we come back to the special shapes of attracting sets that distinguish some systems from others in terms of the states frequented—and the temporal structure of flows on this manifold (Huys et al., 2014).

## Phenomenal transparency and the counterfactual me

Under the premise of deep temporal models, one necessarily encounters a counterfactual depth or richness, in virtue of the fact that the further one goes into the future, the greater the number of possible outcomes. This can be seen easily by picturing a generative model of the future as a deep decision tree with multiple leaves on the future horizon (Huys et al., 2012; Solway and Botvinick, 2015; Keramati et al., 2016). This plurality or richness is considered by some to be a hallmark of consciousness (Seth, 2014b; Palmer et al., 2015)—and has some interesting implications. First, it brings us back to the process of selection as something that underwrites conscious processing. This follows because the different ways in which the world could unfold depend upon my action now and in the future. This means that I have to select one particular course of action (because I can only do one thing at one time). In turn, this requires a selection among competing counterfactual hypotheses about the future that will determine my course of action—a selection that is often compared to Bayesian model selection or, possibly, averaging (FitzGerald et al., 2014). This selection collapses a portfolio of counterfactual possibilities into a chosen course of action. The very existence of this requisite selection implies a choice and (in a rather superficial fashion^8^) mandates free will. In short, if we entail generative models with temporal depth:

*“We must believe in free will, we have no choice.”* (Isaac Bashevis Singer)

The notion of counterfactual richness deserves some discussion. Anil Seth offers a compelling analysis of phenomenological objecthood and counterfactual richness:

*“[O]n the relationship between perceptual presence and objecthood, I recognize a distinction between the “world revealing” presence of phenomenological objecthood, and the experience of “absence of presence” or “phenomenal unreality.” Here I propose that world-revealing presence (objecthood) depends on counterfactually rich predictive models that are necessarily hierarchically deep, whereas phenomenal unreality arises when active inference fails to unmix causes “in the world” from those that depend on the perceiver*” (Seth, 2015, p. 1).

It may be that “counterfactual” is used here in the slightly broader sense of alternative or competing hypotheses about the current state of affairs that explain my sensations—and could indeed run counter to the sensory evidence at hand. In contrast, I use counterfactual in the limited sense of relating to—or expressing—what has not *yet* happened. In this sense, counterfactual beliefs pertain to the future consequences of action and necessarily entail temporal depth.

The second implication is that counterfactual hypotheses about “what could be” equips us with the remarkable capacity to entertain “what if” beliefs about the world—in particular, my active engagement with the lived world. This has some interesting implications, if we consider that this affords the opportunity for little (and big) thought experiments. For example, “what would happen if I did that?” This provides an interesting take on the sorts of thought experiments that underlie philosophical “zombies” and the “hard problem” (Chalmers, 1995). These constructs rely upon “what if” questions; such as “what is the quintessential difference between a conscious me and a non-conscious me?” On the current argument, the very fact that these questions can be posed speaks to the capacity to entertain “what if” hypotheses; rendering hard questions of this sort an emergent property of generative models with counterfactual (future) outcomes. In short, the key difference between a conscious and non-conscious me is that the non-conscious me would not be able to formulate a “hard problem”; quite simply because I could not entertain a thought experiment.

So what lends the counterfactual, what if, hypotheses a life of their own? Why are they not dissipated by sensory evidence in the same way perceptual hypotheses are selected on the basis of sensation to constitute our percepts and (presumably) qualitative experience? In the terminology of Metzinger, why do high-level counterfactual hypotheses lose a phenomenal transparency (Metzinger, 2003); enabling them to be manipulated by mental action (Metzinger, 2013). In other words, how can we account for the loss of transparency; i.e., a phenomenal opacity that allows us to experience percepts in a way that is not inherent in their qualitative attributes^9^. This is a really interesting question that may be addressed in terms of hierarchical generative models. This follows from the fact that a deep generative model will usually possess a hierarchical structure, with separation of timescales over hierarchical levels (Friston et al., 2017b). A deep model of this sort generates narratives at the highest level that provide prior constraints on faster narratives or flows (i.e., counterfactual trajectories) at lower levels. The very existence of hierarchical generative models implies the loss of phenomenal transparency (or the remarkable capacity for opacity) in the following sense: in physical systems that perform hierarchical Bayesian inference—in accord with the Bayesian mechanics of random dynamical systems—the belief propagation between levels rests upon sufficient statistics. In other words, the beliefs (i.e., probability distributions) are not, in themselves, propagated or shared among the levels; only the statistics or parameters of those beliefs are available to higher levels. Conversely, the influence of descending messages constitutes an effect (of sufficient statistics) on beliefs encoded at lower levels. This means there is an opportunity for “mental action”; i.e., belief updating that does not entail overt action. As soon as there is the opportunity for mental action on beliefs we have, by definition, the capacity for opacity. This sort of argument may provide a useful framework to understand the nature of phenomenal transparency in a hierarchically nested inference process—in a way that is grounded in the neural code (Wiese, 2017). Please see the contribution from Jakub Limanowski (this Research Topic) for a more considered treatment.

## Self and others

Finally, there is one aspect of self-awareness that deserves a special mention. This is the very notion or hypothesis of self *per se*. Many arguments in this setting turn to interoceptive inference and a physiologically *embodied* account of how we infer ourselves to engender a minimal sense of selfhood (Limanowski and Blankenburg, 2013; Seth, 2013; Barrett and Simmons, 2015; Fotopoulou and Tsakiris, 2017b). This is an important dimension to any discussion of self-consciousness; especially when considering selfhood as quintessentially embodied. It is a perspective that gracefully ties in emotions, affect and selfhood under the rubric of interoceptive inference; namely, furnishing plausible explanations for my “gut feelings,” my “pain”—and other sensorial consequences of my interoceptive and autonomic states of being. This is nicely captured by Apps and Tsakiris (2014), who present an account of the neural and computational basis of self-recognition, under the free-energy principle:

*“In this account one's body is processed in a Bayesian manner as the most likely to be ‘me’.”* (Apps and Tsakiris, 2014, p. 85)

There are many engaging aspects to interoceptive inference that speak to the relationship between affect and the embodied self (and others). Some notable contributions over the past years include (Seth et al., 2011; Ainley et al., 2012, 2016; Barrett and Satpute, 2013; Limanowski and Blankenburg, 2013; Seth, 2013, 2014a; Barrett and Simmons, 2015; Seth and Friston, 2016; Stephan et al., 2016; Fotopoulou and Tsakiris, 2017b). A less interoceptive-centric conception of self is pursued in Hohwy and Michael (2017), where self is considered as a hypothesis or explanation that seamlessly explains experience over multiple timescales and modalities. On this view, the “self” is constituted by deeply hidden causes that transcend our forward or generative models of introception:

*“A balanced self-model of endogenous causes may be paraphrased in simple terms as a theory or narrative that appeals to regularities or plotlines at different, interlocked time scales. Such a theory or narrative can be seen as an answer to the question: which kind of agent am* !?” (Hohwy and Michael, 2017, p. 9)

Here, I wanted to focus on the simple observation that in order to talk about the self, our generative models must entertain a distinction between self and non-self—and between self and other. This has been nicely pursued in a developmental setting in terms of dyadic interactions between infants and [m]others (Fotopoulou and Tsakiris, 2017b). From the perspective of Bayesian mechanics, if I exist in a world that is populated with other creatures like me, then I will come to learn this fundamental state of affairs: see for example (Friston and Frith, 2015). In other words, most of my generative model is concerned with modeling you, under the assumption that you are a “creature like me.” Under this hypothesis, the sorts of thought experiments that lead to philosophical “zombies” and the “hard problem” become much more plausible. For example, how would I know whether you are conscious (like me) or not? The basic point here is that the very notion of self-consciousness presupposes that there is an alternative (non-self) consciousness. However, would the distinction between self-consciousness and consciousness have any meaning in the absence of a distinction between self and other—or indeed self and non-self? This begs the interesting question: would a creature that does not have theory of mind need to entertain the hypothesis of self-consciousness—in the sense that a virus cannot contemplate the “hard problem”?

The idea here is that possessing a generative model that can distinguish between self and another is necessary for self-consciousness. As noted by one of my reviewers, this sort of generative model underwrites theory of mind and mentalizing; e.g., (Buckner and Carroll, 2007; Palmer et al., 2015; Hamilton and Lind, 2016; Fotopoulou and Tsakiris, 2017a). The (active inference) imperatives that underlie these generative models also speak to simulation theories of mind reading (Gallese and Goldman, 1998; Kilner et al., 2007)—suggesting a formal link between self-consciousness and consciousness of others.

## Different sorts of consciousness

Does consciousness as (active) inference make any sense practically? I would submit it does. From a psychiatric perspective, altered states of consciousness come in two flavors. There can be a change in the level of consciousness; for example, during sleep, and anaesthesia and coma. Alternatively there can be altered conscious states of the sort associated with psychiatric syndromes and the effects of psychotropic (or psychedelic) drugs. In terms of the current thesis, levels of consciousness speak directly to the enactive aspects of inference above. Put simply, the hallmark of reduced levels of consciousness is an absence of responsiveness (Gosseries et al., 2014). Try to imagine someone who is not conscious but acts in response to stimulation. The only responses one can elicit are reflexes that reflect minimization of surprise in the “here and now.” This suggests a mapping between the level of consciousness and the (temporal) thickness of inference about the proximate future (and past). Interestingly, studies of subjects in minimally conscious states often rely on imaginal or simulated (i.e., counterfactual) activities; such as playing tennis (Owen et al., 2006). In our daily lives, it also suggests that this thickness or depth^10^ waxes and wanes with the sleep wake cycle—as we fire up our hierarchical predictive processing machinery during the day and do the (statistical) housekeeping at night: see (Hobson and Friston, 2014) for more discussion. On this view, loss of consciousness occurs whenever our generative models lose their “thickness” and become as “thin” as a viruses.

As a psychiatrist, I am drawn to the notion of altered conscious states as altered inference for several reasons. Key among these is the ability to understand the signs and symptoms of psychiatric disorder as false inference. For example, in classical statistics, there are two types of false inference; *false positives* and *false negatives*. False positives correspond to inferring something is there when it is not; like hallucinations, delusions and other false ideation in psychosis (e.g., schizophrenia). Conversely, false negatives are when one fails to infer something when it is there; i.e., a failure to recognize something or to entertain impossible ambiguities (e.g., “who are you,” “am I the right way up,” and so on). This translates clinically into disorientation and various forms of agnosia that characterize dementias and other organic psychosyndromes. From a practical point of view, this is a useful perspective because the neuronal machinery behind active inference and predictive processing is becoming increasingly transparent—pointing to the (usually neuromodulatory) pathophysiology that underwrites false inference, psychopathology and, by induction, altered states of (self) consciousness (Corlett et al., 2011; Adams et al., 2013; Powers et al., 2017).

## Conclusion

There are many issues that have been glossed over in this brief treatment—and many that could be unpacked further; particularly in the neurosciences. In the past few years, the appreciation that expected surprise (i.e., uncertainty) figures so centrally in active inference has led to a number of interesting insights—and links with established (psychological and computational) constructs. In brief, the imperative to minimize surprise *per se* can be usefully linked to a variety of global brain theories; including, reinforcement learning (Daw et al., 2005; Dayan and Daw, 2008; Botvinick et al., 2009), optimal control theory (Erez and Todorov, 2012; Kappen et al., 2012), expected utility theory in economics (Gold and Shadlen, 2007; Bossaerts and Murawski, 2015), the principles of maximum efficiency and minimum redundancy (Barlow, 1974; Optican and Richmond, 1987; Linsker, 1990), the Bayesian brain hypothesis (Knill and Pouget, 2004) and predictive coding (Mumford, 1992; Rao and Ballard, 1999; Michael and De Bruin, 2015)—and so on. However, these formulations do not necessarily imply any consciousness processing. For example, exactly the same predictive coding principles—used to explain perceptual synthesis in the visual system—can be used to simulate pattern formation and morphogenesis at the level of single cells (Friston et al., 2015a).

However, when we start to look more closely at the minimization of expected surprise; namely, the resolution of uncertainty, things get much more interesting. For example, expected surprise (a.k.a. expected free energy) neatly separates among a number of dimensions (Friston et al., 2015b). The two most important include a separation into epistemic and pragmatic value; also known as intrinsic and extrinsic value. The epistemic part scores the resolution of uncertainty (e.g., turning on the lights in a dark room), while the pragmatic part involves avoiding costly surprises (e.g., looking directly at the sun). Another interesting way of carving expected surprise is in terms of *ambiguity* and *risk* that have some close connections with economic formulations of optimal decision-making. It is this distinction between simply minimizing surprise and expected surprise (uncertainty) that we have focused on in distinguishing conscious from non-conscious inference. Whether this is useful or not remains to be seen—but at least it brings a bit of physics to the table.

In conclusion, we have gone—fairly rapidly—through the following arguments. First, if we want to talk about living things, we have to identify the necessary behaviors and properties those things must possess. This is fairly easy to do by noting that living implies the existence of an attracting set of states that are frequented in a characteristic way. This implies the existence of a Lyapunov function that is formally identical to surprise (or self-information) in information theory and Bayesian model evidence in statistics. This means that all (biological) processes can be construed as an inference process from evolution right through to conscious processing. If this is the case, then what at point do we invoke consciousness? The proposal on offer here is that the self-evidencing has a temporal thickness and depth, which underwrites inferences about the counterfactual consequences of action. This necessarily lends (active) inference a purposeful and self-centered aspect that has the hallmarks of consciousness (and necessarily implies self-consciousness because I am the author of my actions). This means that the defining feature of consciousness is the self-consciousness entailed by active inference—especially when you are part of my generative model.

## Author contributions

The author confirms being the sole contributor of this work and approved it for publication.

### Conflict of interest statement

The author declares that the research was conducted in the absence of any commercial or financial relationships that could be construed as a potential conflict of interest.

## Footnotes

^1^Although this trick should not be taken too seriously, it can be thought-provoking. For example, I was challenged by my editors to apply it to the following question: Example: “*Can the subjectivity of consciousness be explained in terms of properties of the brain*?” vs. “*Can the subjectivity of evolution be explained in terms of properties of the phenotype*?” This transposition immediately raises ontological questions about subjectivity. The phenotype is certainly subject to evolution and the phenotype is the subject of selective pressure. Furthermore, the evolution of a nematode is not the evolution of a Neanderthal (i.e., your subjectivity is not my subjectivity). Is a phenotype subjectively aware it is evolving—and if so, what does “awareness” mean?—and so on.

^2^Unless, of course, the answer is “consciousness is for evolution”—see Frith (2010).

^3^Strictly speaking, the second law of thermodynamics does not apply in this instance, because the (living) systems we are talking about are thermodynamically open. However, there are generalizations of the second law (known as fluctuation theorems) that apply to open systems far from equilibrium. These fluctuation theorems suggest that the probability of entropy decreasing becomes vanishingly small with time scale (Evans and Searles, 2002; Seifert, 2012). Self-evidencing appears to fly in the face of these theorems—or does it? Self-evidencing (the principle of least surprise or free energy) is an example of Hamilton's principle of least Action. This is because the time average of a Lyapunov (energy) function is Hamiltonian Action. When the Lyapunov function is self-information Hamilton's Action becomes Shannon's entropy. This means that self organization and self-evidencing may be perfectly natural, and lawful. So why do we think it is so remarkable? Perhaps the answer lies in our ability to entertain counterfactual beliefs—something we will return to at the end of this article.

^4^We have skipped over some interesting technical considerations here. Specifically, we have assumed that a process or system can be distinguished from its environment (e.g., a heat bath in thermodynamics). Technically, this induces something called a *Markov blanket* that is constituted by a subset of states that provide a statistical separation between states that are part of the system (internal states) and those states that are outside the system (external states): see Figure 1. Note that this separation is permeable. In other words, it is purely a statistical separation that allows for internal states to be coupled vicariously to external states, through the Markov blanket: see Friston (2013) and Clark (2017). In thermodynamic terms, this means the system is open.

^5^In this paper, thick and deep models are used synonymously. In machine learning, policies are often optimized using deep tree searches that necessarily have a “thick” temporal span.

^6^Note that there is a fundamental distinction between planning a sequence of actions (i.e., sequential policy optimization) under a deep temporal model and simply pursuing a state-action policy. Technically, state-action policies prescribe the best action from any currently inferred state—and are the sort of policies optimized under the Bellman optimality policy (e.g., reinforcement learning and optimal control theory). The behavior of many biological systems; e.g., *E. coli*, and pigeons can be described in terms of state-action policies; however, here, we are referring to sequential policy optimization of the sort required for planning as inference: Botvinick and Toussaint (2012). This form of (active) inference means that what we (infer ourselves to) do depends on *beliefs about* states of the world in the future—not states of the world *per se*. see for further discussion (Friston et al., 2015b).

^7^The equivalence between expected surprise and uncertainty appeals to the weak ergodicity of the systems we are considering.

^8^I mean this in the sense that model or policy selection is a process that is enacted by an agent, through autonomous (free energy minimizing) dynamics, which could—of course—be subpersonal.

^9^E.g., “I have a ringing noise in my ears,” as opposed to “I can hear something ringing.”

^10^Technically, in hierarchical generative models there is usually a one-to-one mapping between the temporal thickness or extent and hierarchical depth. In other words, higher levels of a hierarchical model generally represent sequences or trajectories with a greater temporal span. This speaks to the intriguing possibility of associating hierarchical depth with temporal thickness; where one can assess the depth of neuronal hierarchies using purely neuroanatomical criteria.
